# Cerebral, ocular, dental, auricular, skeletal anomalies (CODAS) syndrome: First case reported in Saudi Arabia

**DOI:** 10.1016/j.radcr.2022.11.085

**Published:** 2023-01-05

**Authors:** Ahmed Hafez Mousa, Hussein Omar Taher, Fawziah Alzaid Al Sharif, Hala Rafat Zulali, Reem Saud Alqufaidi, Yasmeen Salah Alsulaiman, Raneem Yasser Gazaz, Mohammed Fouad Alamer, Elsayed Mohamed Mehena

**Affiliations:** a College of Medicine and Surgery, Batterjee Medical College, P.O. Box 6231, Jeddah 21442, Saudi Arabia; b Department of Pediatrics, Saudi German Hospital, Jeddah, Saudi Arabia; c Children's Health Center, Department of Pediatrics, International Medical Center, Jeddah, Saudi Arabia; d Medical Research Institute, University of Alexandria, Alexandria, Egypt; e Medicine Department, Al-Rayan Colleges, Madinah, Saudi Arabia; f Medicine Department, Fakeeh College for Medical Sciences, Jeddah, Saudi Arabia

**Keywords:** Codas syndrome, Developmental disabilities, Rehabilitation

## Abstract

CODAS syndrome (cerebral, ocular, dental, auricular, skeletal anomalies) is a rare autosomal recessive inherited multisystemic disease that carries an incidence rate of less than 1 in 1,000,000 children worldwide. It has an infancy, neonatal age of onset, characterized by deformities of the central nervous system, eyes, ears, teeth, and skeleton. A 1-year-old female of non-consanguineous parents, first time presented to our pediatrics clinic on November 6, 2021 when she was 4 months of age with developmental delay, as the patient could not support her head and made no eye contact on examination. Microcephaly was observed. She had a positive family history; her sister died at the age of 3 days with microcephaly and diaphragmatic hernia. We recommend that a wider range of centers to get encouraged to report cases of CODAS they might encounter due to the lack of sufficient amounts if solid literature on the topic. To our knowledge, this is the first case to be reported in the literature from Saudi Arabia.

## Introduction

CODAS syndrome (cerebral, ocular, dental, auricular, skeletal anomalies) is a rare autosomal recessive inherited multisystemic disease that carries an incidence rate of less than 1 in 1,000,000 children worldwide. It has an infancy, neonatal age of onset, characterized by deformities of the central nervous system, eyes, ears, teeth, and skeleton [1]. It was first disclosed on a 4-year-old girl [2]. Specific constellation of anatomical findings should make identification of patients simple. the anomalies in this condition are craniofacial anomalies, median nasal groove, dental-aberrant cusp morphology and delayed eruption, auricular-malformations of the external ear, hearing loss, ocular-cataracts, ptosis, skeletal-spondyloepiphyseal, short stature, delayed epiphyseal ossification, metaphyseal hip dysplasia, malformed helices, vertebral coronal clefts, and intellectual disability [3]. Due to a lack of curative medical treatment, there is currently no effective treatment for this rare hereditary disorder but rehabilitation could play a significant role in genetic illness treatment [4]. LonP1 is a mitochondrial matrix protease whose selective substrate specificity is necessary for preserving mitochondrial homeostasis. Previous reports have linked pathogenic LonP1 mutations to CODAS syndrome [5].

## Clinical presentation

A 1-year-old female of non-consanguineous parents, first time presented to our pediatrics clinic on November 6, 2021 when she was 4 months of age with developmental delay, as the patient could not support her head and made no eye contact on examination. Microcephaly was observed. She had a positive family history; her sister died at the age of 3 days with microcephaly and diaphragmatic hernia.

**Fig. 1:**
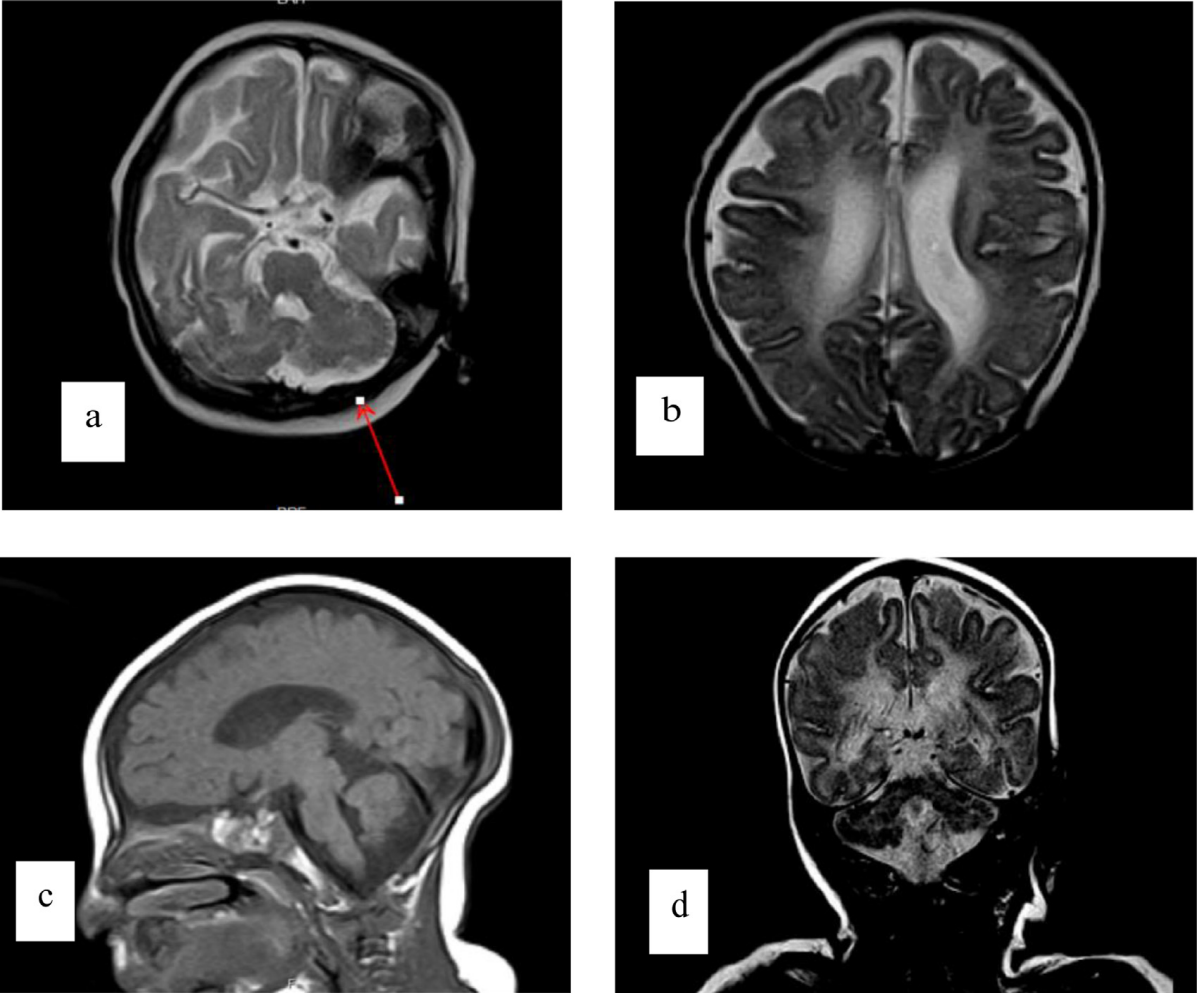
(a, b) Axial T2, (c) sagittal T1, (d) coronal T2. Axial T2 (a, b) at different levels. Sagittal T1 (c) and coronal T2 (d). Small-sized cranium. There is paucity of periventricular white matter mainly along peri-trigonal regions, with undulation of lateral aspect of both lateral ventricles. There is large posterior fossa with bilateral cerebellar hemisphere hypoplasia notably in the left cerebellar hemisphere (arrow in image a) with enlargement of the retro-cerebellar sub-arachnoid space that communicate with the fourth ventricle. There is no evidence of pontine or brainstem hypoplasia. No vermian hypoplasia and myelination is age appropriate.

On examination, body weight was 4.95 kg (below third centile), her height was 59 cm (25th centile), and her head circumference was 37.5 cm (below third centile) indicating microcephaly. The patient was set on Levetiracetam 0.5 g for 10 days. Brain MRI/MRA was ordered, MRI revealed a paucity of periventricular white matter mainly along peri-trigonal regions, with the undulation of lateral margins of both lateral ventricles, representing periventricular leukomalacia. Large posterior fossa, bilateral cerebellar hemisphere hypoplasia with enlargement of the retro-cerebellar sub-arachnoid space that communicates with the fourth ventricle, consistent with the dandy walker variant with no pontine, brainstem, or vermian hypoplasia (Fig. 1). Unremarkable MRA of the brain, with no flow-limiting stenosis, aneurysmal formation, occlusion, or dissection.

On November 16, 2021, the patient came for a follow-up. Levetiracetam was stopped and risperidone 0.25 mg/mL syrup 100 mL X 1 was prescribed for 2 months.

On January 11, 2022, the patient visited the clinic with mild mental retardation, significant impairment of behavior, localization-related focal and partial symptomatic epilepsy, and epileptic syndromes with simple partial seizures. She showed delayed milestones. The patient was planned for EEG. Chloral hydrate 250.00 mg/5 mL was prescribed at bedtime as the mother explained that the patient is having difficulty sleeping. Her brain awake digital EEG record showed the following (Table 1):

**Table 1:** Awake EEG spikes.

Theta	4-5 Hz, low medium voltage, irregular, intermixed diffuse.
Delta	2 Hz, medium voltage, polymorphic.
Artifacts	Some muscle and movement artifacts were noted.
Photic	No driving or activating effects.
Interpretation	This EEG shows a mild diffuse nonspecific distribution of electrical cortical rhythms. No epileptiform discharges were noted during this EEG.

On March 22, 2022, her body weight was 6.2 kg (below third centile) and her height was 64.5 cm (fifth centile), indicating failure to thrive. We started levetiracetam 100 mg/mL 300ML SYRUP 1 mg/mL BID for 3 months. EEG showed active spikes (Table 2).

**Table 2:** EEG spikes.

Theta	4-5 Hz, low-medium voltage, irregular, intermixed diffusely in sleep.
Delta	2 Hz, medium voltage, polymorphic, diffuse.
Sleep	No vertical wave or sleep spindles were noted.
Photic	No driving or activating effects.
Spikes	Focal spikes were noted involving O1.
interpretation	This EEG shows active left occipital epileptiform discharges superimposed on diffuse background slowing.

On December 4, 2021, whole exome sequencing was performed for this patient and her parents identified an interesting missense variant in the LONP1 gene, LONP1 (NM_004793.4):c.2263C>G (p.Arg755Gly), Chr19(GRCh37):g.5694455G>C.

## Discussion

CODAS syndrome is a very rare disease characterized by instinctive features including developmental delay, craniofacial anomalies, ptosis, cataracts, median nasal groove, hearing loss, delayed tooth eruption, short stature, metaphyseal hip dysplasia, delayed epiphyseal ossification, and vertebral coronal clefts [6]. CODAS was first described in 1991 as a distinct constellation of growth retardation, craniofacial abnormalities, cataracts, ptosis, mid-nasal sulcus, delayed teething, abnormal crown morphology, heterochromic torsion malformations, hearing loss, short stature, epithelial retardation, hip dysplasia, and metaphyseal vertebrae [7]. In the previous 20 years, only 4 seemingly sporadic cases have been reported, demonstrating that the entire phenotypic is actually extremely rare [8]. In 1 CODAS case, 12-year-old Japanese male patients, doctors detected in LONP1, a compound heterozygous mutation [8]. A paternally inherited frameshift mutation (p.Ser100Glnfs*46) was found in one allele and a maternally inherited missense mutation (p.Arg786Trp) in the other allele was predicted to be pathogenic using web-based prediction methods. The preservation of mitochondrial homeostasis depends on the selective substrate specificity of LonP1, a mitochondrial matrix protease. Previous studies have suggested that the complex multisystemic and developmental condition CODAS syndrome is caused by recessively inherited, pathogenic abnormalities in LonP1 gene. It is interesting to note that despite the well-known substantial clinical variety of conventional mitochondrial disease presentations, the skeletal and dental characteristics linked to CODAS syndrome are pathognomonic. By the age of 6 months, dense nuclear cataracts can be observed. A single infant's fundi were reported as normal at 1 month old, yet the child's vision was described as 20/200 at 2 years old [9]. Pediatric neurologists and physiatrists (who specialize in children's rehabilitation) find it tough to provide an effective treatment for genetic diseases. Given the challenges in selecting the right type of gene therapy and when to initiate it, deciding the treatment and management must be based on scant information to alleviate specific indications and symptoms of the condition [10]. Therefore, according to the patient's symptoms, pediatric neurologists and physiatrists advise rehabilitative treatment. Although there hasn't been any comprehensive research on rehabilitation, patients should be encouraged to engage in physical activity to enhance their quality of life. It is important that patient's parents must help their kids to become contributing members of society, and independent adults later on in life [9].

Our patient presented with microcephaly, Dandy-Walker malformation, no head support, no eye contact, positive family history (her sister died at the age of 3 days with microcephaly and diaphragmatic hernia), and of a non-consanguineous parents. After performing whole exome sequencing for the patient and her parents, it identified an interesting missense variant in the LONP1 gene (Table 3).

**Table 3:** MAF (minor allele frequency) based on gnomAD data.

Summary of variant(s) identified in the patient and her parents.								
Gene and transcript	cDNA change	Amino acid change	Molecular consequence	Zygosity			MAF*	Mode of inheritance
LONP1 NM_004793.4	c.2263C>G	p.Arg755Gly	Missense	Index	Mother	Father	0.0007%	Autosomal Recessive AR
				Hom	Het	Het		

Hom, homozygous; Het, heterozygous.

A posterior fossa defect known as the Dandy-Walker malformation (DWM) or syndrome is characterized by vermis agenesis or hypoplasia and cystic expansion of the fourth ventricle, which pushes the tentorium and torcula upward. At the time of diagnosis, the majority of individuals have hydrocephalus [11]. While some individuals may present with a range of comorbidities that contribute to an early diagnosis, others may remain clinically asymptomatic for years. The Luschka and Magendie foramina's atresia, which results in an expansion of the fourth ventricle and vermian hypoplasia, was once thought to be the primary cause of DWM [11]. Recent research, however, points to the possibility that DWM is caused by developmental anomalies that disrupt the rhombencephalon's roof and result in varying degrees of vermian hypoplasia and cystic growth [12]. The failure of the fourth ventricle foramina fenestration, which results in an enlarged Blake's pouch and compresses the vermis, and the arrest of vermian development are 2 separate pathophysiological causes that might contribute to the development of this complicated deformity. DWM is a congenital brain anomaly and over 85% of cases are discovered before the age of 1 year. Five researchers have reported that the incidence of hydrocephalus was between 86% and 94% in numerous prior studies [13]. Prenatal regular sonography throughout the antenatal period has helped some obstetricians discover this malformation [14]. According to 7 different studies following prenatal ultrasonographic examinations, 80% of individuals with DWM showed no signs of hydrocephalus, suggesting that hydrocephalus may develop during the neonatal period [15].

## Conclusion

Unfortunately, most disorders with a genetic background, as in CODAS, cannot be cured. Such disorders affect various body systems with a wide spectrum of severity and impairment. We recommend that a wider range of centers to get encouraged to report cases of CODAS they might encounter due to the lack of sufficient amounts if solid literature on the topic.

## Patient consent

Written consent was obtained from the patients parents for usage of the information presented in the case.
